# Notes and News

**Published:** 1897-05-29

**Authors:** 


### NOTES AND NEWS.

(Collected and Communicated.)

H.R.H. Princess Louise, Marchioness of Lorne, has promised to open the Convalescent Home at Cranbrook, Kent, in July, given by Mr. Passmore Edwards to the Metropolitan Hospital, N.E.

The "Past" v. "Present" cricket and lawn tennis matches of the amalgamated clubs of St. Bartholomew's Hospital will take place on June 16th, at Winchmore Hill, after which, at 7.45, there will be a dinner at the Holborn Restaurant.

An American contemporary, commenting upon the proposed anti-vivisectionist hospital for London, says that the idea is as absurd as if a vegetarian set up as a butcher, seeing that the patients will be treated by anti-vivisectionists according to principles discovered through vivisection.

By the will of Lady Victoria Wellesley a large number of charitable institutions benefit to a considerable extent, amongst the number being the Hospital and Home for Incurable Children, Maida Vale, £500; the Seaford Convalescent Hospital, £500; St. George's Hospital, £500; the Royal Free Hospital, £500; the Great Ormond Street Children's Hospital, £500; the Cancer Hospital, Fulham Road, £500; Samaritan Free Hospital for Women and Children, £500; the National Hospital for the Paralysed and Epileptic, £800; the National Society for the Prevention of Cruelty to Children, £1,000; and many others.

The Staffordshire General Infirmary is again in occupation. It has undergone complete reconstruction. Previously the building consisted of a single block with a central corridor, the wards opening out of either side. At the end of the corridor was a wing for infectious cases. The central portion has been retained, and converted into an administrative block, containing also the nurses' quarters. The wards are new, and are situated in pavilions joining the administration block by open corridors. Four wards contain fourteen beds each. A children's ward contains twelve beds, and there are three isolation wards with two beds, and three with one bed. Two day-rooms have been provided. An operating theatre, out-patients' department, and other offices are also added. The cost of the reconstruction amounts to £20,000.

The foundation-stone of the New Paisley Infirmary has been laid by Mrs. Stewart Clark.

A handsome anonymous donation of £1,000 has been given by "H. L. R." to St. Thomas's Hospital for the purpose of endowing a bed.

The annual distribution of prizes to the students of Charing Cross Hospital Medical School will take place on Wednesday, June 2nd, at four o'clock, at the Medical School, Field-Marshal Sir J. Lintorn Simmons, G.C.B., in the chair.

Dr. George Stoker's Oxygen Home in Fitzroy Square was opened by the Princess Louise on May 19th. The home has been established to carry on the treatment of ulcers and wounds by oxygen as advocated and practised by Dr. Stoker. There were already thirty-six cases in the home on the occasion of the opening ceremony. Attached to the home is a well-fitted laboratory.

We are glad to learn that the Commemoration Bible and Prayer Book, which contains one of the Hospital Stamps specially issued in connection with the Prince of Wales's Hospital Fund, has proved such a success that the Oxford University Press, who are issuing the Prayer Book, have had to purchase a further 20,000 one shilling Hospital Stamps for insertion therein, the supply that they had at first secured proving insufficient.

The visit of the Prince of Wales to Oxford has benefited the Radcliffe Infirmary through the generosity of local firms, who erected stands from which the procession was viewed, and who devoted the proceeds to the Infirmary. Thus, from Messrs. Elliston and Cavell upwards of £21 were received, and from Messrs. J. Zacharias £4 10s. The example of these firms might with advantage be followed elsewhere during the festive jubilee time when processions abound.

It is good news to learn that all the land needed for the reconstruction and enlargement of University College Hospital has been secured, and the demolition of one-fourth of the houses on the intended site is being commenced this week, though this work will in no way interfere with the carrying on of the hospital. In accordance with Sir J. Blundell Maple's special desire, a much finer entrance has been arranged for the out-patients, so that they may have every comfort while waiting.

It is a curious outcome of all the fuss that has been made about direct representation of the profession on the General Medical Council to find Dr. Rentoul saying that he could not well attend the May session *for more than one day*, and asking, therefore, that a particular day should be fixed for the discussion of an elaborate notice of motion which he wished to have placed on the agenda. But it is still more interesting to find that, when he discovers that his pet motion cannot be so placed, he writes to the papers to say that he sees no practical use in his attending the May session at all! We cannot pretend to agree very often with Mr. George Brown, but we certainly agree with him when he says that in acting thus Dr. Rentoul "only imperfectly realises his position as a direct representative."

